# BHLHE41 suppresses MCF‐7 cell invasion via MAPK/JNK pathway

**DOI:** 10.1111/jcmm.15033

**Published:** 2020-02-19

**Authors:** Di Zhang, Qin Zheng, Chen Wang, Na Zhao, Yang Liu, Enhua Wang

**Affiliations:** ^1^ Department of Pathology The First Affiliated Hospital China Medical University Shenyang China; ^2^ Department of Pathology College of Basic Medical Sciences China Medical University Shenyang China

**Keywords:** BHLHE41, breast cancer, invasion, JNK signalling pathway, tight junction

## Abstract

Deregulation of the basic helix‐loop‐helix family member e41 (BHLHE41) has been characterized as a marker of progression of several cancers. In this study, we aimed to explore the mechanism by which BHLHE41 regulates the invasion of breast cancer cells. BHLHE41 suppresses, whereas the silencing of BHLHE41 promotes tumour invasion of both MCF‐7 and MDA‐MB‐231 cells. Meanwhile, BHLHE41 down‐regulated the transcription and translation of SNAI1, SNAI2, VIM and CDH2, and up‐regulated those of CLDN1, CLDN4 and CDH1. Reporter assay indicated that silencing of BHLHE41 dramatically activated the MAPK/JNK signalling pathway in MCF‐7 cell line and the hypoxia signalling pathway in MDA‐MB‐231 cell line. Furthermore, silencing of BHLHE41 activated the MAPK/JNK signalling pathway by up‐regulating phosphorylated JNK and failed to affect the expression of HIF‐1 alpha in MCF‐7 cells. After blocking the MAPK/JNK signalling pathway by specific inhibitor SP600125, silencing of BHLHE41 failed to promote tumour cell invasion. These results suggest that BHLHE41 facilitates MCF‐7 cell invasion mainly via the activation of MAPK/JNK signalling pathway. In conclusion, although BHLHE41 suppresses tumour invasion in MCF‐7 and MDA‐MB‐231 cell lines, the specific regulatory mechanisms may be different.

## 1 INTRODUCTION

Basic helix‐loop‐helix family member e41 (BHLHE41, also known as SHARP1, DEC2 and BHLHB3) is mapped to human chromosome 12p12.1. It serves an important role in regulating cell differentiation, maintaining circadian rhythm, apoptosis, hypoxia and immune response.^1^, ^2^, ^3^, ^4^, ^5^, ^6^, ^7^, ^8^, ^9^, ^10^, ^11^ The major functions of BHLHE41 are divided into two categories: circadian and non‐circadian regulation. For the former, BHLHE41 protein re‐enters the nucleus after transcription and translation and competes with CLOCK‐BMAL1 heterodimer for E‐Box element binding (through competitive inhibition). BHLHE41 functions as a suppressor of circadian rhythm regulation in this process.^11^, ^12^ For non‐circadian regulation, BHLHE41 is implicated in multiple other pathways, such as BCR, notch, hypoxia, ERK/NF‐kappaB and PI3K/Akt pathways.^3^, ^6^, ^8^, ^13^, ^14^, ^15^

Dysregulation of BHLHE41 transcription levels has been characterized as a marker of the progression of several cancers.^8^, ^16^, ^17^, ^18^, ^19^, ^20^, ^21^, ^22^, ^23^ Recent studies indicate that BHLHE41 is involved in regulating tumour migration and invasion.^5^, ^6^, ^19^, ^24^ Asanoma et al^19^ demonstrated that BHLHE41 suppressed tumour invasion and metastasis in endometrial cancer by inhibiting TWIST1 transcription. Li et al^6^ pointed out that BHLHE41 suppressed tumour proliferation and metastasis by regulating ERK/NF‐kappaB pathway in gastric cancer. BHLHE41 was demonstrated to be a crucial regulator of the invasive and metastatic phenotype in triple‐negative breast cancer and suppressed metastasis by promoting the degradation of hypoxia‐inducible factors in MDA‐MB‐231 cells.^8^ Thus far, most researchers believe that BHLHE41 plays a role in suppressing cell invasion and metastasis in tumour progression. The detailed mechanisms, however, were not always consistently demonstrated in the above studies.^6^, ^8^, ^19^ Therefore, further work is needed to understand the mechanism by which BHLHE41 regulates cell invasion and metastasis.

Montagner et al^8^ demonstrated that BHLHE41 suppressed invasion and metastasis by promoting the degradation of HIF‐1α in triple‐negative breast cancer cell line MDA‐MB‐231. They also pointed out that BHLHE41 expression correlated with the higher metastatic molecular phenotype (triple‐negative breast cancer). It is still unknown whether and by what mechanism BHLHE41 equally suppresses invasion and metastasis in MCF‐7 cells (luminal A phenotype). This study aimed to answer these questions. By transwell assay, we found that BHLHE41 suppressed cell migration and invasion, while BHLHE41 silencing by siRNA promoted them in MCF‐7 and MDA‐MB‐231 cells. We also analysed the variations of epithelial‐mesenchymal transition (EMT)‐associated factors by quantitative real‐time PCR (qRT‐PCR) and Western blot (WB) analysis. The results indicated that BHLHE41 silencing by siRNA promoted cell invasion through inducing EMT. We further screened multi‐signalling pathways by reporter assay. BHLHE41 had a greater effect on MAPK‐JNK signalling pathway than on the hypoxia pathway in MCF‐7 cells. After blocking MAPK‐JNK signalling pathway by an inhibitor, we found that BHLHE41 silencing by siRNA failed to promote tumour cell invasion. Our results demonstrated that BHLHE41 suppressed invasion not only in MDA‐MB‐231 cells but also in MCF‐7 cells. In contrast to that in the MDA‐MB‐231 cell line, BHLHE41 suppressed invasion via MAPK/JNK signalling pathway in MCF‐7 cell line.

## 2 MATERIALS AND METHODS

### 2.1 Cell culture

The MCF‐7 and MDA‐MB‐231 cell line was obtained from the Shanghai Cell Bank (Shanghai, China). Both cell lines were authenticated by DNA profiling by short tandem repeat (STR). Following that, the cell samples were frozen and individual aliquots were cultured typically for analysis within ten passages. The cells were routinely grown in DMEM (Invitrogen) plus 10% foetal bovine serum (FBS; Invitrogen), 100 μg/mL streptomycin (Sigma) and 100 IU/mL penicillin (Sigma), and passaged every other day using trypsin (0.25%, Invitrogen).

### 2.2 Matrigel invasion assay

Cell invasion assays were performed using 24‐well Transwell chambers (8‐μm pore‐sized; Costar). The inserts were coated with 20 μL Matrigel (1:3 dilution; BD Bioscience). Forty‐eight hours after transfection, the cells were trypsinized and resuspended in 100 μL of a serum‐free medium at a concentration of 3 × 10^5^ cells. The cells were then added to the upper chamber of Transwell plates, and 10% FBS was placed in the lower chamber as a chemoattractant. After an 18‐hour incubation period, the cells that had migrated through the Matrigel‐coated filter were fixed with 4% paraformaldehyde, stained with haematoxylin. The cells on the lower surface of the filters were counted under a light microscope in 10 randomly selected fields at 40× magnification.

### 2.3 qRT‐PCR

The total RNA was extracted from the cells and isolated using an RNeasy RNA isolation kit (QIAGEN). First‐strand cDNA samples were synthesized from the total RNA (1 µg) by using ReverTra Ace (TOYOBO). RT‐PCR was performed using an aliquot of the first‐strand cDNA as a template under standard conditions with Taq DNA polymerase (QIAGEN). Real‐time PCR was performed using the SYBR Green Master Mix (Applied Biosystems). The relative expression levels of the target genes were calculated using a △△CT method after normalizing with the housekeeping gene, 18 S. The sequence information used is shown in Table 1.

**Table 1:** Primers used for the qRT‐PCR analysis

Target gene	Accession No.	Sequence of forward primer	Sequence of reverse primer	Amplicon (bp)
BHLHE41	NM_030762	5′‐CGCCCATTCAGTCCGACTT‐3′	5′‐CGGGAGAGGTATTGCAAGACTT‐3′	78
CLDN1	NM_021101	5′‐TCTGGCTATTTTAGTTGCCACAG‐3′	5′‐AGAGAGCCTGACCAAATTCGT‐3′	107
CLDN4	NM_001305	5′‐TGGGGCTACAGGTAATGGG‐3′	5′‐GGTCTGCGAGGTGACAATGTT‐3′	125
SNAI1	NM_005985	5′‐TCGGAAGCCTAACTACAGCGA‐3′	5′‐AGATGAGCATTGGCAGCGAG‐3′	140
SNAI2	NM_003068	5′‐CGAACTGGACACACATACAGTG‐3′	5′‐CTGAGGATCTCTGGTTGTGGT‐3′	87
CDH2	NM_001792	5′‐TCAGGCGTCTGTAGAGGCTT‐3′	5′‐ATGCACATCCTTCGATAAGACTG‐3′	94
VIM	NM_003380	5′‐GACGCCATCAACACCGAGTT‐3′	5′‐CTTTGTCGTTGGTTAGCTGGT‐3′	238
CDH1	NM_004360	5′‐CGAGAGCTACACGTTCACGG‐3′	5′‐GGGTGTCGAGGGAAAAATAGG‐3′	119
RNA18S		5′‐GTAACCCGTTGAACCCCATT‐3′	5′‐CCATCCAATCGGTAGTAGCG‐3′	150

### 2.4 Western blot (WB)

The total protein extraction was performed using a lysis buffer (Pierce). The total protein concentration in the extract was estimated by the Bradford method.^12^ A total of 15 μg of protein per sample were separated via 15%, 10% or 8% SDS‐PAGE, and transferred onto polyvinylidene fluoride membranes (PVDF; Millipore). The membranes were incubated overnight at 4°C with the following primary antibodies: BHLHE41(1:500,Abcam), BHLHE40 (1:100, Sigma), GAPDH (1:2000, Sigma), His‐tag (1:1000, CWBio), Myc‐tag (1:1000, ImmunoWay), CLDN1/Claudin‐1 (1:500, Invitrogen), CLDN4/Claudin‐4 (1:500, Invitrogen), SNAI1/snail (1:500, Cell Signaling Technology), SNAI2/slug (1:500, Cell Signaling Technology), VIM/Vimentin (1:1000, BD Biosciences), CDH2/N‐cadherin (1:1000, BD Biosciences) and CDH1/E‐cadherin (1:500; ProteinTech Group, Inc). The membranes were washed and were treated for 2 hours at 37°C with peroxidase‐conjugated antimouse or anti‐rabbit IgG (Santa Cruz Biotechnology). The bound proteins were visualized using electrochemiluminescence (Pierce) and detected with a bio‐imaging system (DNR Bio‐Imaging Systems).

### 2.5 Co‐immunoprecipitation (Co‐IP)

The whole‐cell lysates from the MCF‐7 cells expressing MYC‐SP1 or interacting with either Flag‐BHLHE40 or His‐BHLHE41 were used for immunoprecipitation. The lysates were immunoprecipitated with either anti‐SP1 or ‐His antibody bound to protein G PLUS‐agarose (Santa Cruz Biotechnology) overnight at 4°C and then separated by SDS‐PAGE. The immunoprecipitated proteins were visualized by WB with an anti‐SP1, ‐Flag or ‐His antibody.

### 2.6 Plasmid transfection and small interfering RNAs

Plasmids pCMV and pCMV‐Myc‐SP1 were purchased from Sino Biological (HG12024‐NM). Plasmid pcDNA3.1, pcDNA3.1‐BHLHE40 and pcDNA3.1‐BHLHE41 were generous gifts from Prof. Kijima, and the efficiency was demonstrated in a previous study.^16^ The plasmid pcDNA3.1‐BHLHE40 was inserted with N‐His‐Flag‐tag, and the plasmid pcDNA3.1‐BHLHE41 was inserted with N‐His‐Myc‐tag as described previously.^25^ The MCF‐7 cells were seeded at a density of 1 × 10^5^ cells per well (35 mm). After 24 hours, the cells were transfected with BHLHE40 or BHLHE41 using Lipofectamine LTX (Invitrogen). The cells were incubated for 24 hours following transfection and subjected to various analyses. NC‐siRNA (sc‐37007) was purchased from Santa Cruz Biotechnology. The sequences for the sense and antisense of three kinds of BHLHE41 siRNA are shown in Table 2. For the siRNA transfection experiments, MCF‐7 and MDA‐MB‐231 cells were seeded at 5 × 10^4^ cells per 35‐mm well. After 24 hours, the siRNA was transfected into the cells using Lipofectamine 3000 reagent (Invitrogen) according to the manufacturer's instructions. The transfected cells were incubated for another 48 hours and subjected to various analyses. The total amount of siRNA was adjusted with the control siRNA.

**Table 2:** The sequences for the sense and antisense BHLHE41 siRNAs

siRNA	Forward primer	Reverse primer
BHLHE41 siRNA#1	5′‐CCGCACAGAUUAAUAGAAATT‐3′	5′‐UUUCUAUUAAUCUGUGCGGTT‐3′
BHLHE41 siRNA#2	5′‐GCUGUAGUCUUGGAAUUAATT‐3′	5′‐UUAAUUCCAAGACUACAGCTT‐3′
BHLHE41 siRNA#3	5′‐CUGGACUAUUCCUCUUUGUTT‐3′	5′‐ACAAAGAGGAAUAGUCCAGTT‐3′

### 2.7 Immunofluorescent staining

The MCF‐7 and MDA‐MB‐231 cells were added to a four‐chamber slide glass and incubated overnight. The cells were then washed with phosphate‐buffered saline (PBS) and fixed with ice‐chilled methanol for 30 minutes and permeabilized with 0.2% Triton X‐100 in PBS for 30 minutes. After washing twice with PBS, the permeabilized cells were placed in 5% normal horse serum in PBS for 30 minutes (to block the nonspecific adsorption of antibodies) before incubating with anti‐CLDN1 (1:50), anti‐CLDN4 (1:50), anti‐BHLHE41 (1:50) and anti‐Myc (1:50) antibody at 4°C overnight. The cells were then incubated for 1 hour with Alexa 488 dye or Alexa 488 dye (Molecular Probes, Inc) conjugated to goat anti‐rabbit IgG antibody, while nuclear staining was performed using Hoechst 33258. The cells were observed using an Olympus IX51 fluorescent microscope (Olympus), and the images were captured with a COOLPIX 5400 camera (Nikon).

### 2.8 Luciferase reporter assay

Cignal Finder Reporter Assay Kit (10‐pathway) (Product no. 336821) was purchased from QIAGEN (China), and the procedure was performed according to the manufacturer's instructions.

### 2.9 Statistical analyses

All analyses were performed using SPSS version 22.0 for Windows (SPSS). Mann‐Whitney *U* test was used to analyse the differences in expression levels and results of invasion assay. The data of the reporter assay and ChIP were analysed using Student's *t* test. The statistical differences between the treatment groups were determined using Super ANOVA and Scheffé's test. A *P*‐value of <.05 was considered statistically significant.

## 3 RESULTS

### 3.1 BHLHE41 silencing by siRNA promotes tumour cell migration and invasion of MCF‐7 cells and MDA‐MB‐231 cells

**Figure 1:**
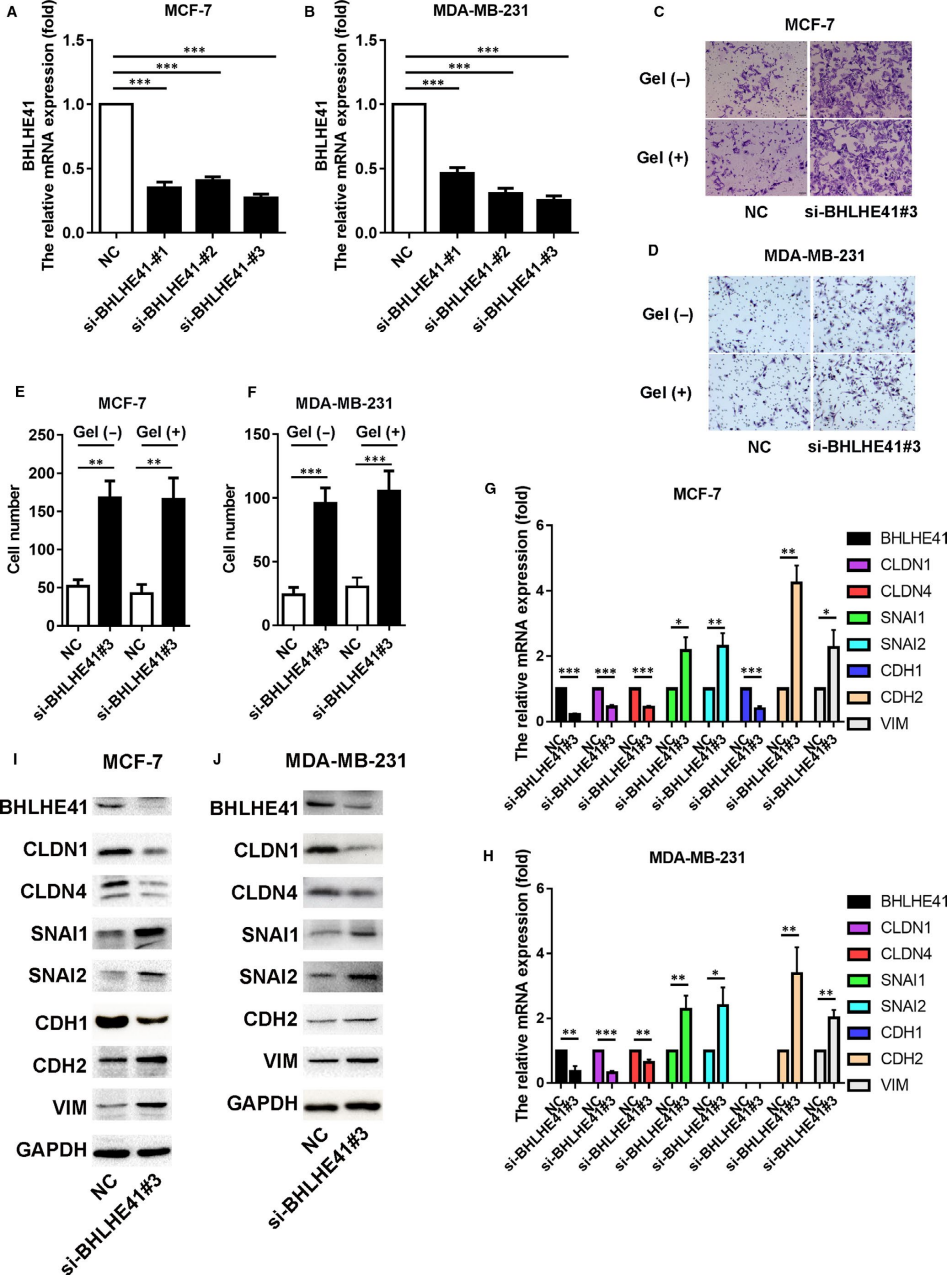
BHLHE41 silencing by siRNA promoted tumour cell migration and invasion of MCF‐7 and MDA‐MB‐231 cells. A, B, Three different siRNAs targeting BHLHE41 were used for the knockdown experiments. The validity of these siRNAs was confirmed by qRT‐PCR. si‐BHLHE41#3 was used in the following knockdown experiments because of the optimal effects. C, D, Transwell assay for assessing cell migration and invasion. As measured by the transwell assay with or without gel cover, BHLHE41 silencing led to a significant increase in the number of migrated cells. E, F, Transwell assays were repeated in triplicate. Ten views were selected for each trial, and the migrated cells were counted for the final statistical analysis. Each value represents the mean ± SE (bars) of three independent experiments, ***P < .001. G, H, Real‐time PCR analyses of BHLHE41, CLDN1, CLDN4, SNAI1, SNAI2, CDH2, VIM and CDH1 mRNA levels in MCF‐7 and MDA‐MB‐231 cells transfected with scrambled siRNA or BHLHE41 siRNA. I, J, WB analysis of the protein expression of BHLHE41, CLDN1, CLDN4, SNAI1, SNAI2, CDH2, VIM and CDH1 after BHLHE41 knockdown in MCF‐7 and MDA‐MB‐231 cells. [G, H: Each value represents the mean ± SE (bars) of at least three independent experiments; *P < .05, **P < .01, ***P < .001; I, J: A representative image of at least three independent experiments with similar results is shown]

MCF‐7 and MDA‐MB‐231 cells were transfected with BHLHE41 siRNA; scrambled siRNA (NC) was used as a control to verify the role of BHLHE41 in invasion of breast cancer cells. Three kinds of siRNAs were prepared for use with BHLHE41. Their efficiencies were confirmed by qRT‐PCR analysis (Figure 1A,B). The siBHLHE41#3 with optimal effect was selected for subsequent experiments. As shown in Figure 1C,D, BHLHE41 silencing by siRNA promoted cell migration and invasion of both MCF‐7 and MDA‐MB‐231 cells. Five views were selected randomly for the analysis. In MCF‐7 cells, the number of migrated cells was 51.8 ± 3.9 and 167.6 ± 10.0 (mean ± SE) in the control group and BHLHE41 siRNA group, respectively. In MDA‐MB‐231 cells, the number of cells were 23.8 ± 2.6 and 95.6 ± 5.5 (mean ± SE), respectively. In the invasion assay, the number of cells that traversed the gel was less in the control group than that in the BHLHE41 siRNA group [42.2 ± 5.3 vs 165.8 ± 12.4 and 30.0 ± 3.4 vs 105.4 ± 7.0 (mean ± SE)] in MCF‐7 and MDA‐MB‐231 cell line (Figure 1E,F).

We further detected the variations of EMT‐ and TJ‐associated genes by qRT‐PCR and WB analysis to explore the role of BHLHE41 in regulating cell invasion. The efficiency of BHLHE41 siRNA was confirmed by qRT‐PCR and WB analysis (Figure 1G‐J). The mRNA and protein levels of CLDN1 and CLDN4 were down‐regulated, while those of SNAI1, SNAI2, VIM and CDH2 were up‐regulated by BHLHE41 siRNA in both MCF‐7 and MDA‐MB‐231 cells (Figure 1G‐J). We also found the mRNA and protein levels of CDH1 were also down‐regulated in MCF‐7 cells, but we failed to detect the expression of CDH1 in MDA‐MB‐231 cells as the latter is an E‐cadherin‐null cell line.

### 3.2 BHLHE41 expression suppressed tumour cell migration and invasion of MCF‐7 cells and MDA‐MB‐231 cells

**Figure 2:**
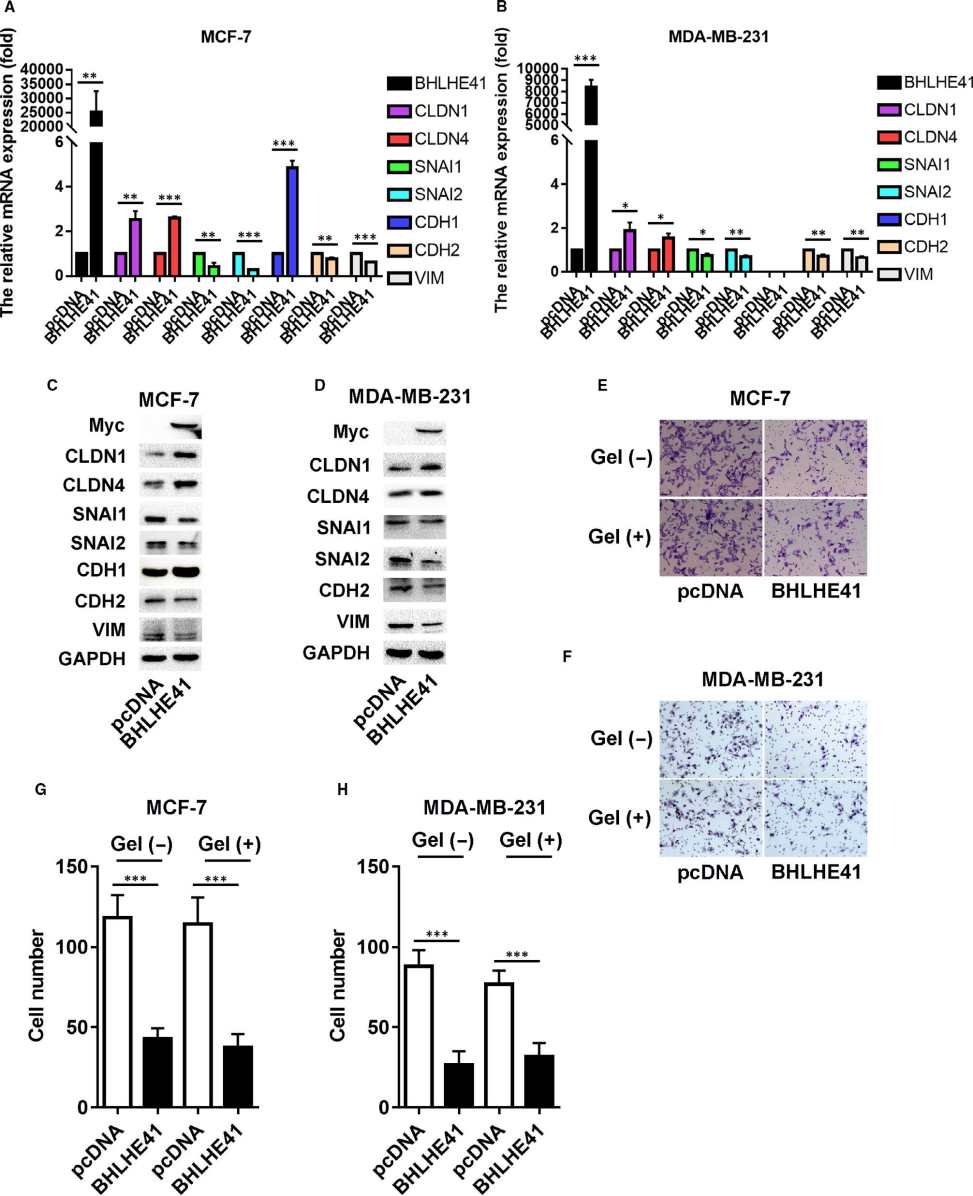
BHLHE41 expression suppressed tumour cell migration and invasion of MCF‐7 cells and MDA‐MB‐231 cells. A, B, The mRNA levels of CLDN1, CLDN4 and CDH1 were up‐regulated, whereas those of SNAI1, SNAI2, VIM and CDH2 were down‐regulated after transfecting with exogenous BHLHE41 in MCF‐7 and MDA‐MB‐231 cells. C, D, WB analysis showed comparable results in the expression of the above genes. E‐H, Transwell assay for assessing cell migration and invasion of MCF‐7 and MDA‐MB‐231. As measured by the transwell assay without or with gel cover, BHLHE41 overexpression led to a significant decrease in the number of migrated and invasion cells. Transwell assays were repeated in triplicate. Ten views were selected for each trial, and the migrated or invasion cells were counted for the final statistical analysis. Each value represents the mean ± SE (bars) of three independent experiments, ***P < .001

We also detect the role of exogenous BHLHE41 in cell invasion. MCF‐7 and MDA‐MB‐231 cell lines were transfected with His‐Myc‐BHLHE41, which used the vector (pcDNA) as a control to verify the function of exogenous BHLHE41. We detected the mRNA and protein expression of EMT and claudins to detect the function of exogenous BHLHE41. The validity of BHLHE41 plasmid was confirmed by qRT‐PCR and WB analysis, respectively (Figure 2A‐D). The mRNA and protein levels of CLDN1, CLDN4 and CDH1 were up‐regulated, whereas those of SNAI1, SNAI2, VIM and CDH2 were down‐regulated after transfecting with exogenous BHLHE41 in MCF‐7 cells (Figure 2A,C). The qRT‐PCR and WB analysis showed comparable results in the expression of the above genes except for CDH1 in MDA‐MB‐231 cell line (Figure 2B,D). As shown in Figure 2E,F, exogenous BHLHE41 inhibited cell migration and invasion in both MCF‐7 and MDA‐MB‐231 cells. In MCF‐7 cell line, the number of migrated cells was 118.2 ± 6.3 and 42.8 ± 3.0 (mean ± SE), while the number of invading cells was 114.2 ± 7.4 and 37.4 ± 3.7 (mean ± SE) in the pcDNA group and BHLHE41 transfected group, respectively. In MDA‐MB‐231 cells, these numbers were 88.0 ± 4.5 and 26.4 ± 3.8 (mean ± SE) and 76.8 ± 3.8 and 31.6 ± 3.8 (mean ± SE), respectively (Figure 2G,H).

### 3.3 BHLHE41 did not affect the cellular location of CLDN1 and CLDN4

**Figure 3:**
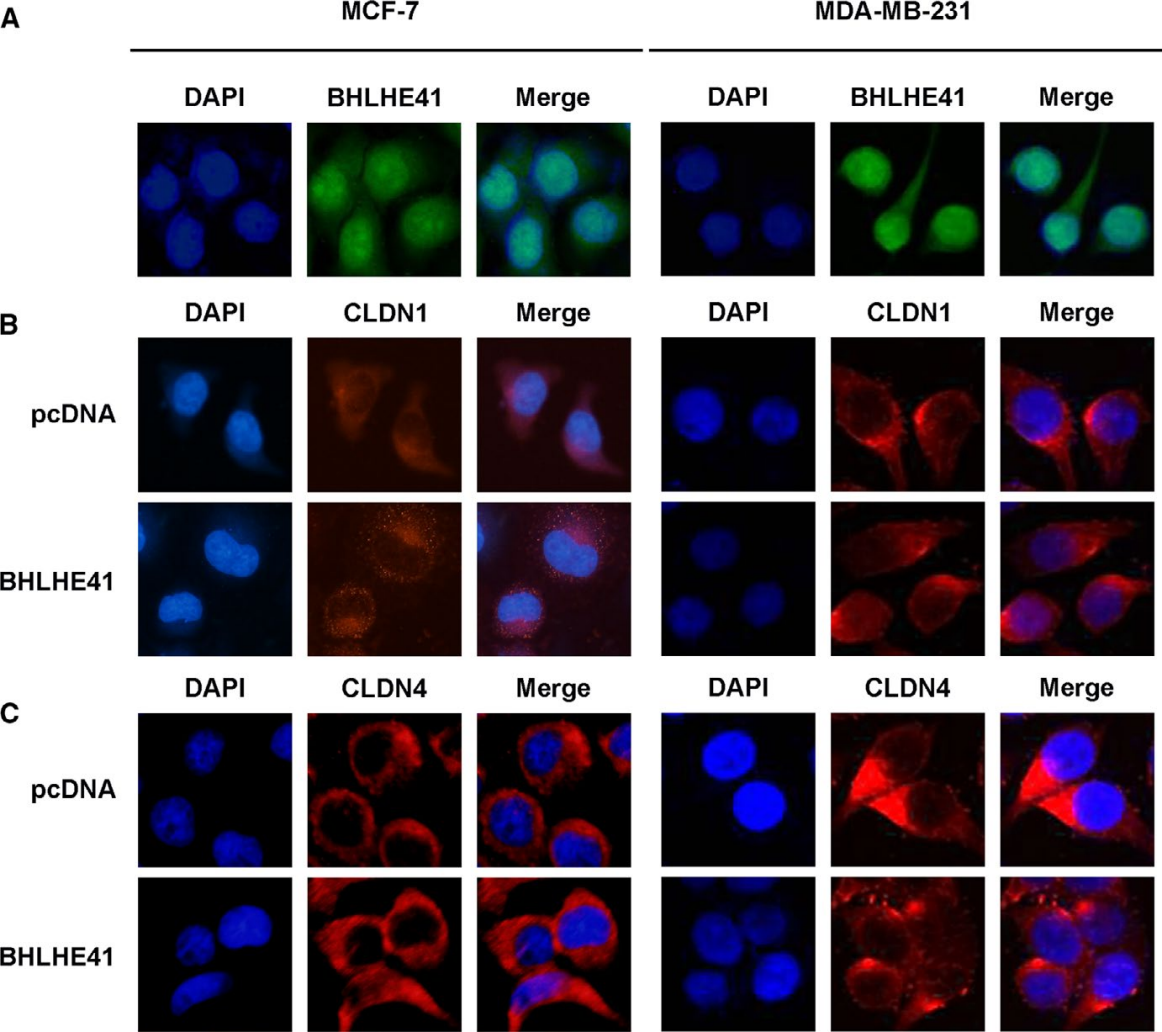
BHLHE41 did not affect the cellular location of CLDN1 and CLDN4. A, BHLHE41 exhibited a nuclear expression pattern in both MCF‐7 and MDA‐MB‐231 cells. B, In MCF‐7 and MDA‐MB‐231 cell line, CLDN1 exhibited a cytoplasmic expression pattern regardless of the BHLHE41 expression level. C, Likewise, the intracellular localization of CLDN4 did not change after BHLHE41 overexpression. A representative image of at least three independent experiments with similar results is shown

We next performed immunofluorescence staining, to investigate whether BHLHE41 affects their intracellular localization of CLDN1 and CLDN4. As shown in Figure 3, BHLHE41 is normally expressed in the nuclear of MCF‐7 and MDA‐MB‐231 cells. Both CLDN1 and CLDN4 exhibited a cytoplasmic expression pattern regardless of the BHLHE41 expression level. In other word, although BHLHE41 might regulate the mRNA and protein expression of CLDN1 and CLDN4, it did not affect their cellular location.

### 3.4 BHLHE41 suppressed MCF‐7 cell invasion via MAPK/JNK signalling pathway

**Figure 4:**
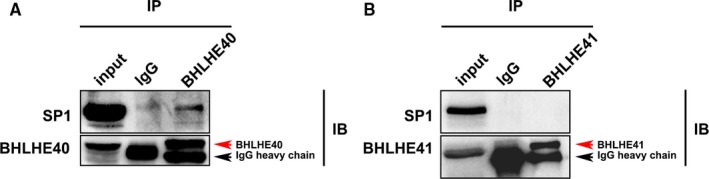
BHLHE41 failed to interact with SP1 in MCF‐7 cells. A, Here, BHLHE40 was used as a positive control. B, BHLHE41 failed to interact with SP1 in MCF‐7 cells. Immunoprecipitation assay using MCF‐7 cells transfecting with Myc‐SP1 and Flag‐BHLHE40 or His‐Myc‐BHLHE41. The cell lysate was immunoprecipitated with antibodies shown at the top and immunoblotted with the antibodies indicated on the left. The red arrows indicate the target bands. The bands indicated by black arrows are IgG heavy chains

We previously reported that BHLHE40, another member of the DEC subfamily, regulated MCF‐7 cell invasion through interaction with SP1 and therefore negatively regulated the transcription of CLDN1. In the current study, we performed Co‐IP to detect whether BHLHE41 could interact with SP1 and play a similar role in regulating CLDN1 transcription. As shown in Figure 4, BHLHE41 failed to interact with SP1 and BHLHE40 was used here as a positive control.

**Figure 5:**
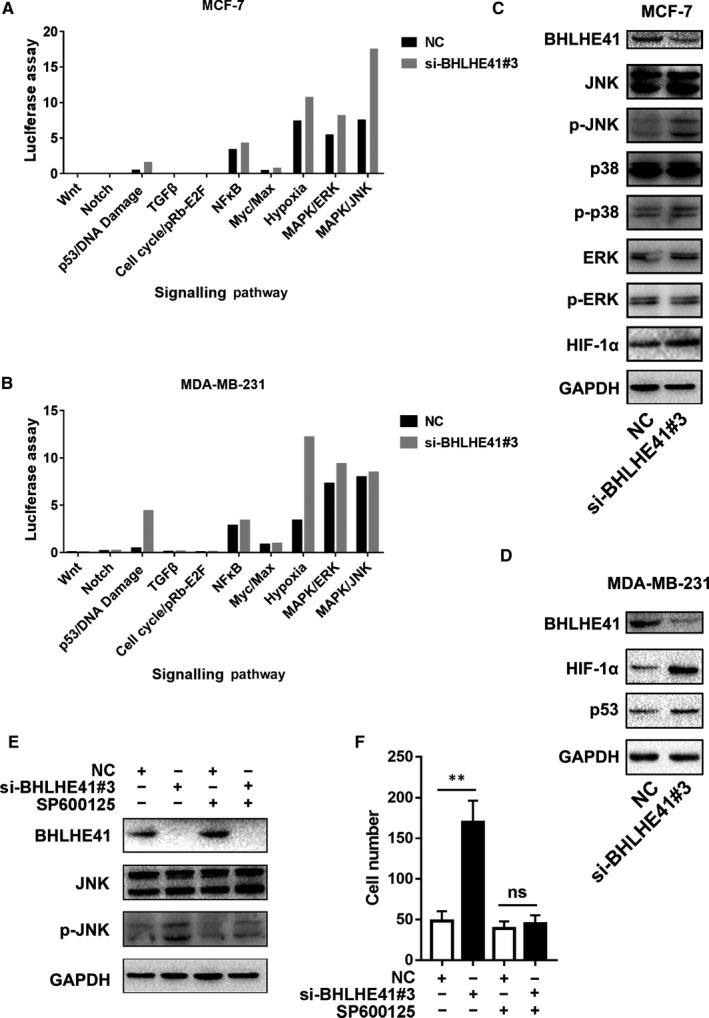
Unlike that in the MDA‐MB‐231 cell line, BHLHE41 suppressed invasion via MAPK/JNK signalling pathway in MCF‐7 cell line. A, Silencing of BHLHE41 activated MAPK/JNK signalling pathway in MCF‐7 cells. B, Silencing of BHLHE41 induced the phosphorylation of JNK and slightly increased HIF‐1α expression, while it failed to induce the phosphorylation of p38 and ERK. C, Silencing of BHLHE41 activated the hypoxia signalling pathway and up‐regulated HIF‐1α expression in MDA‐MB‐231 cells. E, After blocking the MAPK/JNK signalling pathway with specific inhibitor SP600125, the phosphorylation of JNK induced by BHLHE41 siRNA was reversed. F, Silencing of BHLHE41 failed to promote invasion of MCF‐7 cells after addition of inhibitor SP600125. Transwell assays were repeated in triplicate. Ten views were selected for each trial, and the migrated or invasion cells were counted for the final statistical analysis. Each value represents the mean ± SE (bars) of three independent experiments, ***P < .001

As BHLHE41 was reported to be involved in the regulation of many signalling pathways, we further screened multi‐signalling pathways by reporter assay in MCF‐7 and MDA‐MB‐231 cells. We silenced BHLHE41 by siRNA to investigate the effects of endogenous BHLHE41 on these signalling pathways. As shown in Figure 5A, we found that NFκB, hypoxia, MAPK/ERK and MAPK/JNK signalling pathways were activated after treatment with BHLHE41 siRNA in MCF‐7 cell line. Among them, the variations of the MAPK/JNK signalling pathway was the most apparent. The other signalling pathways, such as Wnt, Notch, p53, TGF‐β, cell cycle/pRb‐E2F and Myc/Max were either unchanged or slightly activated. However, the results of MDA‐MB‐231 cell line showed that the variations of hypoxia and p53 signalling pathway were the most apparent, while that of the MAPK/JNK signalling pathway was slightly activated (Figure 5B).

We further performed WB analysis to verify the status of NFκB, hypoxia, MAPK/ERK and MAPK/JNK signalling pathway after treatment with BHLHE41 siRNA. As shown in Figure 5C, phosphorylated JNK was obviously up‐regulated after treatment with BHLHE41 siRNA in MCF‐7 cells, which indicated the activate state of MAPK/JNK signalling pathway. The variations of phosphorylation status and expression of target genes in other pathways were less remarkable. HIF‐1α was slightly up‐regulated, phosphorylated ERK and p38 were almost unchanged. On the contrary, HIF‐1α was dramatically up‐regulated, while the phosphorylated ERK and p38 were almost unchanged after transfecting with BHLHE41 siRNA in an MDA‐MB‐231 cell line (Figure 5D).

To further investigate whether BHLHE41 suppressed the invasion of MCF‐7 cell line via MAPK/JNK signalling pathway, we blocked MAPK/JNK signalling pathway with a specific inhibitor SP600125 and subsequently treated MCF‐7 cells with BHLHE41 siRNA. As shown in WB analysis (Figure 5E), the induction of phosphorylated JNK by BHLHE41 siRNA was reversed. Meanwhile, silencing of BHLHE41 by siRNA could not promote cell invasion any further (Figure 5F).

## 4 DISCUSSION

BHLHE41 is a member of the DEC subfamily within the basic bHLH protein gene family. There are two members within the DEC subfamily, BHLHE40/DEC1 and BHLHE41/DEC2.^5^ Members of this subfamily are involved in regulating various biological phenomena, such as circadian rhythm, cell proliferation, apoptosis, hypoxia and immune responses.^1^, ^2^, ^3^, ^4^, ^5^, ^6^, ^7^, ^8^, ^9^, ^10^, ^11^ A few recent studies have shown that BHLHE40 and BHLHE41 are involved in regulating cell invasion and metastasis.^5^, ^6^, ^19^, ^24^, ^25^, ^26^, ^27^ However, the underlying mechanisms are unclear. We previously reported that BHLHE40 regulates CLDN1 transcription and MCF‐7 cell invasion through binding with sp1.^25^ In the current study, we aimed to explore whether BHLHE41 regulates cell invasion in the same manner as those found in the previous studies.

Our transwell results indicated that BHLHE41 suppressed invasion in both MCF‐7 and MDA‐MB231 cells. This is consistent with the results obtained by other researchers.^6^, ^8^, ^19^ They found that BHLHE41 suppressed cell invasion and metastasis in triple‐negative breast cancer, ovarian cancer and gastric cancer.^6^, ^8^, ^19^ These results indicate that BHLHE41 functions as a suppressor of cell invasion in various cancers. Our data show that BHLHE41 and BHLHE40 play the opposite role in invasion, at least, in breast cancer cells (MCF‐7 and MDA‐MB‐231). In fact, this discrepancy between the performance of BHLHE40 and BHLHE41 is not uncommon.^9^, ^28^, ^29^, ^30^ This paradox was also observed in the regulation of apoptosis and cell proliferation. A possible explanation is that BHLHE40 may negatively regulate the transcription of BHLHE41 and vice versa.^30^ Another explanation is the diversity in protein structure, that is, BHLHE41 has a Gla/Gla‐rich region in its C‐terminal.^5^ In addition, we found that BHLHE41 failed to interact with sp1 and therefore could not form a transcriptional complex as BHLHE40 did. This result indicates that BHLHE41 regulates cell invasion in a different manner as compared to that of BHLHE40.

We screened multiple pathways by reporter assay to find the underlying mechanism of BHLHE41. Interestingly, the activated signalling pathway induced by BHLHE41 is not the same as that in MCF‐7 and MDA‐MB‐231 cell line. Silencing of BHLHE41 by siRNA induced the activation of the hypoxia signalling pathway in the MDA‐MB‐231 cell line and induced the activation of the MAPK‐JNK signalling pathway in MCF‐7 cell line. Montagner et al^8^ previously demonstrated that BHLHE41 suppressed invasion of MDA‐MB‐231 cells by promoting HIF‐1α degradation. BHLHE41^low^/HIF‐1α^high^ phenotype is reported to be positively correlated with lymph node metastasis and triple‐negative molecular subtype of breast cancer. Consistent with their results, we found that silencing of BHLHE41 could activate hypoxia signalling pathway and up‐regulate HIF‐1α expression and protein level in MDA‐MB‐231 cells. On the contrary, silencing of BHLHE41 by siRNA induced phosphorylation of JNK and, therefore, activated MAPK/JNK signalling pathway in MCF‐7 cells. After the addition of a specific inhibitor, silencing of BHLHE41 failed to induce the phosphorylation of JNK and could not continue to promote cell invasion. Our results indicate that BHLHE41 may play a similar role in regulating cell invasion in breast cancers, but the exact pathway may be different according to different molecular subtypes.

MAPK/JNK signalling pathway was reported to be involved in regulating EMT. Phosphorylated JNK may also affect tight junction (TJ) through regulation of claudins.^31^, ^32^, ^33^, ^34^ Our qRT‐PCR and WB results further confirmed this phenomenon. As shown in Figure 1, silencing of BHLHE41 by siRNA might promote EMT and affect TJ through up‐regulating SNAI1/snail, SNAI2/slug and CDH2/N‐cadherin, while down‐regulating CDH1/E‐cadherin, CLDN1/claudin‐1 and CLDN4/claudin‐4. Taken together, BHLHE41 suppresses invasion by inhibiting EMT via MAPK/JNK signalling pathway in MCF‐7 cells. Thus, to conclude, BHLHE41 suppresses invasion of breast cancer cells, but the detailed regulatory pathways may be different according to different cell types.

## CONFLICT OF INTEREST

The authors declare no conflict of interest.

## AUTHOR CONTRIBUTIONS

Yang Liu and Enhua Wang conceived and designed the experiments; Di Zhang, Qin Zheng, Chen Wang and Na Zhao performed the experiments; Di Zhang, Yang Liu and Enhua wrote the paper.

## Data Availability

The data that support the findings of this study are available from the corresponding author upon reasonable request.
